# Parental ADHD knowledge and medical visit status of school-aged children in Shanghai

**DOI:** 10.1186/s13034-024-00780-6

**Published:** 2024-07-22

**Authors:** Xirui Ma, Yuanyuan Lin, Wenjie Yan, Zhijuan Jin, Yiwen Zhang

**Affiliations:** Department of Developmental and Behavioral Pediatrics, National Children’s Medical Center, Shanghai Children’s Medical Center, School of Medicine, Shanghai Jiao Tong University, Shanghai, China

**Keywords:** Attention deficit disorder with hyperactivity, Child, China, Mental Health Services, Social stigma

## Abstract

**Introduction:**

The diagnosis and care of children and adolescents with neurodevelopmental disorders presents a public health crisis in China. Attention deficit hyperactivity disorder (ADHD) is one of the most frequent conditions. Many Chinese children and adolescents with ADHD are underdiagnosed and undertreated. This study aimed to evaluate the awareness and attitude parents have about ADHD, and investigated potential factors influencing ADHD medical visit status among school-aged children in Shanghai.

**Methods:**

A random cluster sampling method was used, and four primary schools in Shanghai were selected. One class was randomly selected from each grade, including students and their parents. Parents completed the Swanson, Nolan, and Pelham Version IV Questionnaire (SNAP-IV) parent form and questionnaire concerning ADHD awareness, knowledge, attitude and status of ADHD medical visit. Descriptive analysis was conducted on the overall results and logistic regression analysis was performed to explore the influencing factors of ADHD medical visit.

**Results:**

We received 617 valid questionnaires. There were 313 boys (50.7%) and 304 girls (49.2%), with a median age of 8 years old (p25 = 7, p75 = 9). 42.4% parents believed they had some knowledge about ADHD, and 73.5% of them thought ADHD was a neurologically based disorder or neurological condition by nature. Parental ADHD information came from the following sources: Internet/TV (*n* = 458, 74.2%), families/friends (*n* = 267, 43.2%), print publication (*n* = 208, 33.7%), psychiatrists/pediatricians (*n* = 192, 31.1%), schools/teachers (*n* = 186, 30.1%) and other ADHD patients (*n* = 48, 7.7%). When children had ADHD-like behaviors, most parents (61.5%) educated children to behave themselves, 59.1% parents tried to get help from psychiatrists/pediatricians, 55.5% of them would ask psychologist for help. In terms of the ADHD prevalence, the SNAP-IV positive screen rate was 4.3% (*n* = 27). Only 33.3% (9/27) of parents went to the hospital for consultation and treatment. Multivariate logistic regression model showed that parental knowledge about ADHD (OR = 13.67, 95%CI: 1.72, 144.39, *P* = 0.01) was significantly correlated with the medical visit. Parents with sufficient knowledge of ADHD tend to visit hospital for help when they thought their children had ADHD related symptoms.

**Conclusions:**

The majority of parents accepted ADHD as a neurodevelopmental disorder by nature, but some parents still had certain misunderstandings about ADHD. The main source of information for parents to obtain information about ADHD was through the TV/Internet. Parents’ perceptions and knowledge were key to whether children received appropriate treatment for their ADHD. However, medical visits to address ADHD among school-aged children were still lower than expected. Government and healthcare institutes should work to improve ADHD public awareness and to help patients and their families gain access to mental health resources.

## Introduction

Attention deficit hyperactivity disorder (ADHD) is a common neurodevelopmental disorder in childhood and adolescence [1]. It is characterized by the core symptoms of hyperactivity and inattention. ADHD is commonly diagnosed in childhood and often persists into adulthood. It is also a risk factor for other mental issues such as defiant and disruptive behaviors, and emotional or other behavioral problems [2–5]. The global prevalence of ADHD is 7.6% in children. In the United States, ADHD affects approximately one in 10 children and adolescents aged 3–17 years [6]. A meta-analysis published in 2017 showed the overall pooled-prevalence of ADHD among Chinese children and adolescents was 6.26% [7]. Chinese society has changed dramatically by industrialization and urbanization over the past four decades and children’s neurodevelopmental disorders have been increasingly recognized as constituting a public health crisis by mental health service providers and policymakers [8, 9].

To provide nationwide epidemiological evidence to support the need for up-to-date mental health services, Li and colleagues conducted the first national-scale psychiatric epidemiological survey of children and adolescents in 2022. They reported the prevalence of ADHD among 6- to 16-year-old Chinese children and adolescents as 6.4% using the Swanson, Nolan, and Pelham Version IV Questionnaire (SNAP-IV) [8].

China has recently initiated the Healthy China Action (HCA) program to promote people’s health. One of its major goals is to promote mental health of the children and adolescents by improving the delivery of the health services [8]. With the developments in pediatric psychiatry and the emergence of the subspecialty of developmental & behavioral pediatrics, the efficiency of diagnosis and treatment of ADHD has been enhanced in developed Chinese cities, such as Shanghai. However, many children are still underdiagnosed and undertreated. One study in 2022 reported that when children were diagnosed with ADHD for the first time, parents showed insufficient knowledge of this disease profile in treatment decision making [10]. It appears that most parents lack sufficient understanding of ADHD, potentially affecting their participation and adherence to therapy. For example, a study found that parents with valid knowledge about ADHD were more accepting of both behavioral and medication treatments. In contrast, those who believed there was a social stigma attached to the diagnosis and who had lacked understanding about ADHD showed greater acceptance of alternative and less effective interventions, such as dietary management [10, 11]. Though much effort has been made in the past few decades to increase therapeutic services, the compliance of children with ADHD to various therapies in China is relatively poor. A systematic review and meta-analysis revealed that among children and adolescents in China, on average only 10% of parents of children with ADHD sought medical consultation, and approximately only one third received medication [7].

Limited studies have reported public perceptions about ADHD in parents, and the associations between parental ADHD awareness and medical visit status in Chinese children. We created a questionnaire to explore the perceptions about ADHD in parents of school-aged children and the reasons why parents delayed or were deterred from seeking help or treatment. We also identified some risk factors which may be responsible for decreasing the ADHD medical visits. We anticipate that the information reported in this study will fill the gap in this regard and will be a valuable reference for family-centered ADHD intervention engagement care and government policymaking.

## Methods

### Participants and data collection

This database was collected in Shanghai, as part of an up-to-date nationwide psychiatric epidemiological survey of school-aged children. Four primary schools, including two key primary schools and two normal primary schools, were randomly chosen in Shanghai. In each of these schools, we randomly picked one class in grades 1 through 5 and asked parents to complete the SNAP-IV and the questionnaire developed for this project. A total of 617 students were included in this survey.

Responders had to be able to read and understand Mandarin and be one of caregivers of their children. The demographic characteristics of children and parents, family social economic status and other basic information were collected. Parents completed the SNAP-IV parent form and an ADHD Awareness Survey & ADHD Diagnosis and Treatment Status Survey.

### Socioeconomic status

Parental education and household income were self-reported. Parental educational levels (the educational level of the responder) were categorized into 5 groups: elementary school and below, middle school, junior college, undergraduate, master and above. Household income per month was divided into 4 categories: ≤8000 RMB (Chinese Yuan), 8001–17,000, 17,001–30,000, ≥ 30,001.

### The Swanson, Nolan, and Pelham Version IV Questionnaire

To assess ADHD symptom, we used the SNAP-IV 18-Item Parent Rating Scale. The scale is an abbreviated version of the SNAP Questionnaire [12]. Items from the Diagnostic and Statistical Manual, Fourth edition (DSM-4) criteria for ADHD are included for the two subsets of symptoms: Inattention and Hyperactivity/Impulsivity. Severity of symptom was rated on a 4-point scale ranging from not at all (scored 0) to very much (scored 3). Questions (items 1–18) summarizing the inattention and hyperactivity/impulsivity domains were used in this survey. The Chinese SNAP-IV Parent-rating scale applied here was generally consistent with the English version with good reliability and validity. The internal consistency, as measured by the Cronbach α, was 0.95 for the total scale. The Cronbach α for the inattention domain and the hyperactivity/impulsivity domain were 0.90 and 0.89 respectively [13].

A positive screening on the SNAP-IV was defined as at least six of nine items of the Inattention with a score of ≥ 2 and/or at least six of nine items of the Hyperactivity/Impulsivity ≥ 2. A positive screen served as an indication for ADHD risk [14].

### ADHD awareness survey questionnaire & ADHD diagnosis and treatment status questionnaire

This questionnaire, consisted of true/false questions and Likert items, had three parts. The first part collected demographic characteristics, including the age and sex of the child, paternal age and maternal age, the relationship between the responder and the child, parental educational levels (the educational level of the responder), weekly time spent with the child and household income. The second part was a parental self-report ADHD Awareness Survey Questionnaire that was used to collect data on parents’ knowledge and attitudes about ADHD. The third part was an ADHD Diagnosis and Treatment Status questionnaire collecting information about treatment preferences and concerns about ADHD treatment strategy, including single-choice and multiple-choice questions.

### Statistical analysis

All data were evaluated with Epidata 3.0. Descriptive analysis was conducted on the overall results and logistic regression analyses were used to identify the influencing factors of ADHD medical visit in children whose parents thought they had ADHD related symptoms. *P* < 0.05 was considered statistically significant. Data analysis was conducted with R language 4.3.0.

### Ethics

All children with their parents joined this study with informed consent. The protocol was approved by the Ethics Committee of the Shanghai Children’s Medical Center, School of Medicine, Shanghai Jiao Tong University (SCMCIRB-K2020118-1). The study was conducted in accordance with the Declaration of Helsinki.

## Results

### Distribution of participants

We distributed questionnaires to 644 participants and received 617 responses. As shown in Table 1,  313 (50.7%) were boys and 304 (49.2%) were girls. The median age of children was 8 (7, 9) years. The median parental ages were 38 (37, 41) years for mothers and 40 (38, 43) for fathers. The majority of parents had undergraduate degrees (*n* = 313, 50.7%) or higher educational level (*n* = 224, 36.3%). Children participating in the survey were equally distributed in all 5 grades. We found that 1.7% (*n* = 11) of the parents reported spending less than 7 hours with their child every week, while 57.2% (*n* = 353) of parents spent more than 36 hours with their child weekly.

**Table 1:** General characteristics of the parents and children who participated in the survey

Indicators	All (*n* = 617)
Age of the child, median (p25, p75)	8 (7, 9)
Paternal age	40 (38, 43)
Maternal age	38 (37, 41)
Sex of the child	
Male	313 (50.7%)
Female	304 (49.2%)
Relationship between the responder and the child	
Father (with blood relationship)	143 (23.1%)
Mother (with blood relationship)	470 (76.1%)
Father (without blood relationship)	0 (0%)
Mother (without blood relationship)	1 (0.1%)
Others	3 (0.4%)
Weekly time spent with the child	
< 7 hours	11 (1.7%)
7–14 hours	39 (6.3%)
15–21 hours	64 (10.3%)
22–28 hours	81 (13.1%)
29–36 hours	69 (11.1%)
> 36 hours	353 (57.2%)
Parental educational level	
Elementary school and below	2 (0.3%)
Middle school	2 (0.3%)
Junior college	76 (12.3%)
Undergraduate	313 (50.7%)
Master and above	224 (36.3%)
Household income	
≤ 8000	87 (14.1%)
8001-17,000	163 (26.4%)
17,001–30,000	168 (27.2%)
≥ 30,001	199 (32.2%)
Grade of the child	
Grade 1	118 (19.1%)
Grade 2	118 (19.1%)
Grade 3	125 (20.2%)
Grade 4	144 (23.3%)
Grade 5	112 (18.1%)

Household income was calculated by months and in RMB (Chinese Yuan)

### Parental access to ADHD information

In terms of access to ADHD information sources, parents reported deriving information about ADHD from the following sources: Internet/TV (*n* = 458, 74.2%), families/friends (*n* = 267, 43.2%), print publication (*n* = 208, 33.7%), psychiatrists/pediatricians (*n* = 192, 31.1%), schools/teachers (*n* = 186, 30.1%) and other ADHD patients (*n* = 48, 7.7%).

### Parental attitudes and behaviors towards ADHD

In terms of ADHD knowledge, the majority of parents (*n* = 262, 42.4%) reported they had some knowledge about ADHD, 15.3% (*n* = 95) parents did not understand what ADHD was, and 39.0% (*n* = 241) of the parents knew a little about ADHD. Only 3.0% (*n* = 19) thought they knew much about ADHD (Table 2).

**Table 2:** Parental knowledge and attitude to ADHD and access for ADHD information

Questions	*n*	Values
Knowledge of ADHD (single choice)	617	
A. No knowledge at all		95 (15.3%)
B. Very limited knowledge		241 (39.0%)
C. Some knowledge		262 (42.4%)
D. Sufficient knowledge		19 (3.0%)
Do parents think ADHD is a neurologically based disorder or neurological condition by nature (multiple choices)	617	
A. Yes		454 (73.5%)
ADHD is not a neurologically based disorder		
B. Trait		80 (12.9%)
C. Lack of willingness to learn		57 (9.2%)
D. Bad habit		114 (18.4%)
E. Behavioral problem		135 (21.8%)
F. Lack of discipline		71 (11.5%)
G. Moral issue		7 (1.1%)
H. Others		11 (1.7%)
ADHD information source for parents (multiple choices)	617	
A. Psychiatrists/pediatricians		192 (31.1%)
B. Families/friends		267 (43.2%)
C. Internet/TV		458 (74.2%)
D. Print publication		208 (33.7%)
E. Schools/teachers		186 (30.1%)
F. Other ADHD patients		48 (7.7%)
How do parents react when the child has ADHD-like behaviors (multiple choices)	617	
A. Teach children to behave themselves		380 (61.5%)
B. Seek help from psychiatrists/pediatricians		365 (59.1%)
C. Ask psychologists for help		343 (55.5%)
D. Ask teachers for help		203 (32.9%)
E. Look for help from families and friends		52 (8.4%)
F. Did not seek any type of help		20 (3.2%)

Almost 3/4 of parents thought ADHD is a neurologically based disorder or neurological condition by nature. Less than 1/4 parents held the view that ADHD was not a neurologically based disorder or neurological condition by nature. Among these, the most common understanding of ADHD was that it is a behavioral problem (21.8%), a bad habit (18.4%), a trait (12.9%), or the result of lack of discipline (11.5%), lack of willingness to learn (9.2%), or a moral issue (1.1%). Detailed information was shown in Table 2.

### Parents reaction and medical visit rate when children had ADHD-like behaviors

As shown in Table 2, when children had ADHD-like behaviors, most parents tried to teach their children to behave themselves (*n* = 380, 61.5%). 59.1% (*n* = 365) of parents sought help from psychiatrists/pediatricians. 55.5% (*n* = 343) asked psychologists for help. 32.9% (*n* = 203) of them asked teachers for help, and 8.4% (*n* = 52) of them looked for help from families and friends. Only 3.2% (*n* = 20) did not seek any type of help.

Among the 617 participants, 27 (4.3%) had a positive screen on the parent-rated SNAP-IV. The lowest SNAP-IV positive rate was in children who were in grade 1 (*n* = 1, 0.1%). The highest rate was in children in grade 4 (*n* = 8, 1.2%). Among the parents of these 27 subjects, 33.3% (9/27) chose to visit a hospital for help. In addition, 34.0% (210/617) parents thought their child had ADHD related symptoms, but among those only 16.2% parents (34/210) chose to visit hospital for help. And 12.4% of them (26/210) were positive screening by parent-rating SNAP-IV (Table 3).

**Table 3:** Parents-rating SNAP-IV results and ADHD related behaviors

Items	*n*	Values
Parent-rating SNAP-IV results	617	
Negative Screen		590 (95.6%)
Grade 1		117 (18.9%)
Grade 2		112 (18.1%)
Grade 3		120 (19.4%)
Grade 4		136 (22.0%)
Grade 5		105 (17.0%)
Positive Screen		27 (4.3%)
Grade 1		1 (0.1%)
Grade 2		6 (0.9%)
Grade 3		5 (0.8%)
Grade 4		8 (1.2%)
Grade 5		7 (1.1%)
**Questions**		
Do parents think their child has one or more ADHD related symptoms (single choice)	617	
A. Yes		210 (34.0%)
SNAP-IV negative screen		184 (29.8%)
SNAP-IV positive screen		26 (4.2%)
B. No		407 (66.0%)
If the child had ADHD related symptoms, would parents visit a clinic (single choice)	210	
A. No visit		176 (83.8%)
B. Visiting general hospitals		7 (3.3%)
C. Visiting children’s hospitals		24 (11.4%)
D. Visiting psychiatric specialized hospitals		3 (1.4%)
Hospital visit status in children with SNAP-IV positive screening (single choice)	27	
A. Do not visit a hospital		18 (66.7%)
B. General hospitals		4 (14.8%)
C. Children’s hospitals		5 (18.5%)
D. Specialized hospitals		0 (0%)

### Analysis of influencing factors related to medical visit

Logistic regression models determined the factors to influence whether parents sought help for their ADHD concerns by visiting a hospital. 210 children of parents who thought their child had ADHD related symptoms were included in the logistic regression. Among these participants, 38 (18.1%) parents had junior college degrees, 104 (49.6%) parents had undergraduate degrees and 68 (32.3%) had master and above degrees. No parents had middle school or elementary school degree. Therefore, the reference of parental educational level was junior college degree in all the regression models. In univariate regression models (Table 4), the results indicated that parents’ knowledge about ADHD (OR = 15.20, 95%CI: 2.37, 133.49, *P* = 0.005) was strongly correlated with the medical visit. This association was still statistically significant in multivariate models (OR = 13.67, 95%CI: 1.72, 144.39, *P* = 0.01, Table 5). Accordingly, parents with sufficient knowledge of ADHD tend to visit hospital for help when they thought their children had ADHD related symptoms. Factors that were found to have no correlation with ADHD medical visit included parental educational level, monthly household income, weekly time spent with the child, and child’s sex and age.

**Table 4:** Factors related to medical visit according to the univariable logistic regression models

Variables	Univariable analysis (*n* = 210)				
	OR (95% CI)	Estimate	SE	*P* value	
Weekly time spent with the child					
< 7 hours	Ref.				
7–14 hours	0.77 (0.06, 18.30)	-0.25	1.30	0.84	
15–21 hours	0.87 (0.09, 19.23)	-0.13	1.23	0.91	
22–28 hours	3.00 (0.43, 60.36)	1.09	1.14	0.33	
29–36 hours	1.47 (0.17, 31.50)	0.38	1.20	0.74	
> 36 hours	1.20 (0.19, 23.40)	0.18	1.10	0.86	
Parental educational level					
Junior college	Ref.				
Undergraduate	0.68 (0.27, 1.82)	-0.38	0.481	0.42	
Master and above	0.64 (0.23, 1.85)	-0.43	0.525	0.40	
Knowledge of ADHD					
No knowledge at all	Ref.				
Very limited knowledge	0.58 (0.16, 2.14)	-0.53	0.637	0.39	
Some knowledge	2.48 (0.91, 8.01)	0.91	0.544	0.09	
Sufficient knowledge	15.20 (2.37, 133.49)	2.72	0.988	0.005**	
Household income					
≤ 8000	Ref.				
8001-17,000	0.84 (0.27, 2.73)	-0.17	0.57	0.76	
17,001–30,000	0.70 (0.22, 2.32)	-0.35	0.58	0.55	
≥ 30,001	1.14 (0.38, 3.62)	0.13	0.56	0.81	
Age of the child	0.94 (0.72, 1.23)	-0.05	0.136	0.66	
Sex of the child					
Male	Ref.				
Female	0.61 (0.26, 1.33)	-0.48	0.40	-	

SE: Std. Error; ‘**’ is *P* < 0.01

**Table 5:** Factors related to medical visit according to the multivariable logistic regression models

Variables	Multivariable analysis (*n* = 210)			
	OR (95% CI)	Estimate	SE	*P* value
Weekly time spent with the child				
< 7 hours	Ref.			
7–14 hours	0.43 (0.02, 11.79)	-0.83	1.42	0.55
15–21 hours	0.67 (0.05, 16.52)	-0.38	1.31	0.76
22–28 hours	1.86 (0.21, 41.93)	0.62	1.24	0.61
29–36 hours	0.93 (0.08, 23.48)	-0.06	1.33	0.96
> 36 hours	0.63 (0.07, 14.03)	-0.45	1.22	0.71
Parental educational level				
Junior college	Ref.			
College	0.56 (0.19, 1.69)	-0.56	0.54	0.29
Master and above	0.40 (0.11, 1.38)	-0.91	0.63	0.14
Knowledge of ADHD				
No knowledge at all	Ref.			
Very limited knowledge	0.52 (0.13, 2.07)	-0.64	0.67	0.34
Some knowledge	2.56 (0.85, 8.94)	0.94	0.58	0.10
Sufficient knowledge	13.67 (1.72, 144.39)	2.61	1.09	0.01*
Household income				
≤ 8000	Ref.			
8001-17,000	1.30 (0.37, 4.81)	0.26	0.64	0.67
17,001–30,000	0.76 (0.20, 2.92)	-0.27	0.66	0.68
≥ 30,001	1.50 (0.38, 6.19)	0.41	0.69	0.55
Age of the child	0.88 (0.64, 1.20)	-0.12	0.15	0.44
Sex of the child				
Male	Ref.			
Female	0.67 (0.26, 1.65)	-0.38	0.46	0.40

SE: Std. Error; ‘*’ is *P* < 0.05

## Discussion

Long-term outcomes of ADHD have been shown to improve by a consistent and standardized treatment model combining with medication and behavior therapy [15]. Parental attitudes have also been found to play a key role in the long-term outcome of ADHD [10]. A few studies have discussed the importance of parental ADHD knowledge and attitudes in children’s prognosis in the US and European countries [10, 16, 17]. Our study found that parents with sufficient knowledge of ADHD tend to visit hospital to seek professional help when parent thought their children had ADHD related symptoms.

The global prevalence of ADHD is 7.6% in children [6]. Li and colleagues conducted an up-to-date nationwide epidemiological survey in 2022. They reported the prevalence of ADHD was 6.4% in Chinese children and adolescents aged 6–16 years [8]. The prevalence of ADHD in East China was 5.42% (95%CI: 4.54–6.38) [7]. In our findings, parents-reported positive rate of SNAP-IV was 4.3%, which was relatively lower than previously reported findings. One reason for this may be that the diagnosis of ADHD in this study was based on a history and observation of signs and symptoms by parents. Epidemiological studies have reported prevalence data on ADHD by using different criteria, including the Diagnostic and Statistical Manual, Fifth edition (DSM-5), the International Classification of Diseases-10 (ICD-10) or the SNAP-IV Questionnaire [7, 8]. Some differences exist between the key points of those tools that might account for part of the inconsistency in results. A study conducted in Northeast China showed the positive screening rate of ADHD was only 2.7% (*n* = 1118) in primary school students by using parent-rating SNAP-IV, which was markedly lower than our results [18]. The socioeconomic level reported in that study was lower than ours. In our results, 27 children were positive on the SNAP-IV. All the parents had undergraduate or higher level degrees. We suggest that parents in our study are more highly educated and have higher income levels, so they may have been more aware of ADHD and more sensitive to whether their children showed ADHD behaviors.

Children with substantial difficulties with hyperactive and inattentive behaviors are at risk of ADHD and may be just under the threshold to meet the criteria for ADHD [6]. When parents have misconceptions about ADHD, they may be conflicted in making decisions regarding advice and care for their children. We found most parents (73.5%) thought ADHD was an existing neurological disorder by nature, which is consistent with studies in Eastern China and Iran [10, 19]. Sometimes parents holding misconceptions about ADHD may tell themselves that once their children get older, they will have more structure and grow out of it. However, in most cases they do not [20].

Parental fear of stigma is another significant barrier to seeking treatment [21]. ADHD symptoms often persist from childhood to adulthood [22]. The rate of persistent ADHD is approximately 15% at 25 years, with a partial remission rate of approximately 65% [23]. Children with ADHD should be treated during childhood to reduce their clinical symptoms and family burden [24]. Parents of children with ADHD are at increased risk for depression, anxiety and stress [25]. The perceptions of courtesy stigma or affiliate stigma is a common problem among parents of children with ADHD. Higher levels of ADHD symptoms may reinforce mother’s blame, and thus reinforce stigma. This may, in turn, strengthen depression, anxiety and negative self-evaluations [26].

In addition, stigma arises from the lack of ADHD awareness and the prejudice about symptom etiology [27]. In our findings, no more than half of parents reported they had knowledge about ADHD. The prejudice about ADHD symptom includes behavioral problem, a bad habit, the result of lack of discipline, lack of willingness to learn, etc. Some parents described a long process of acceptance to ADHD symptoms over years from onset of these ‘bad habit’ to when they realized these symptoms were indicative of ADHD [20]. Improving ADHD public awareness could play a key role in the long-term outcome of ADHD.

Researchers suggested a need for a deeper investigation about the sources of information that contributed to knowledge and misconceptions of ADHD [28]. Most parents in our study acquired information through the Internet and TV, which was consistently with local and Western studies [18, 29, 30]. For most parents in our study, their information came from social media platforms like WeChat [10]. A Canadian study reported content quality about ADHD was poor on the social media. On TikTok, approximately half of the videos about ADHD they analyzed were misleading [31]. In our study, only one third of parents received information from psychiatrists/pediatricians (31.1%) and schools/teachers (30.1%). Misleading videos about ADHD are being widely disseminated. Therefore, government and healthcare institutes need provide parents more accessible ADHD information and better ADHD training through hospitals and schools. One limitation to this study was our failure to investigate whether parents received correct or incorrect information from different sources.

## Conclusions

This study found that the majority of parents accepted ADHD as a neurological disorder by nature, but some parents still had certain misunderstandings about ADHD. The main source for parents to obtain information about ADHD is the TV/Internet. Previous research has shown that parent’s perceptions and knowledge were key to ADHD prognosis, but the best source of good information about ADHD– from healthcare providers via medical visits– was noted to be less frequently obtained compared with less reliable sources, such as the TV/internet. Government and healthcare institutes should work to improve ADHD public awareness and to improve access to mental health resources.

## Data Availability

No datasets were generated or analysed during the current study.
